# Intermittent double-layer closure for full-thickness defect after super minimally invasive stepwise resection of early rectal cancer

**DOI:** 10.1055/a-2665-7244

**Published:** 2025-08-22

**Authors:** Shuai Tian, Qianqian Chen, Yaoqian Yuan, Kunming Lv, Enqiang Linghu

**Affiliations:** 1 104607 Department of Gastroenterology, The First Medical Center, Chinese PLA General Hospital, Beijing, China; 2 651943 Department of Gastroenterology, 970 Hospital of the PLA Joint Logistic Support Force, Yantai, China

Endoscopic local resection is increasingly regarded as a feasible alternative to radical surgery for specific early rectal cancer patients, preserving organ function and reducing the incidence of complications 1 2 . However, the safe closure of large full-thickness defects after local full-thickness resection remains challenging 3 . This report describes a novel solution – the application of super minimally invasive stepwise full-thickness resection combined with an intermittent double-layer closure technique. Stepwise full-thickness resection is a new technique that combines endoscopic submucosal dissection (ESD) with direct full-thickness resection, using the exposed muscular layer after ESD for the first layer of closure; then, the second layer of closure is performed from mucosa to mucosa.

The patient was an 81-year-old female admitted for early rectal cancer. After a comprehensive assessment, she underwent super minimally invasive stepwise full-thickness resection and the defect was successfully closed using the intermittent double-layer closure technique ( Fig. 1 , Video 1 ). The mucosal layer around the lesion was circumferentially resected and trimmed to the submucosa ( Fig. 1 **a** ). The intrinsic muscular layer was fully exposed using the tissue clip―rubber band-assisted traction technique ( Fig. 1 **b** ). Under a clear field of vision, the location of the central cancer focus was determined, and the muscular layer was incised until the rectal mesenteric fat tissue was visible. The full-thickness resection was performed at this site, leaving a defect of approximately 2.5 cm × 2.2 cm, with the full-thickness defect being about 2.0 cm × 1.6 cm ( Fig. 1 **c, d** ). After hemostasis with electrocautery and cauterization of the wound edge, several tissue clips were used to align and suture the muscular layer to the muscular layer ( Fig. 1 **e** ). The wound surface narrowed after the muscular layer closure, and then, the mucosal layer to mucosal layer was aligned and sutured in the same way, ultimately achieving a tight closure of the wound ( Fig. 1 **f** ). The lesion size was recorded from the serosal layer and the mucosal layer, respectively ( Fig. 1 **g, h** ). The patient had no postoperative bleeding or delayed perforation and was discharged after one week of recovery.

**Fig. 1:**
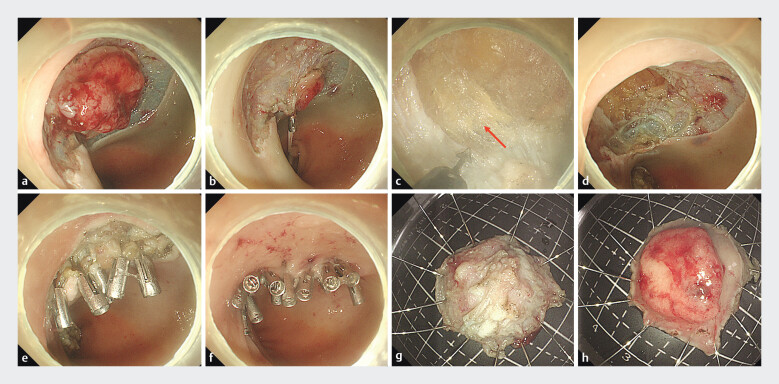
The technical steps of intermittent double-layer closure of the wound after super minimally invasive stepwise full-thickness resection for early rectal cancer. **a** The mucosal layer was circumferentially incised with an electrocautery knife, and part of the submucosal layer was stripped. **b** Tissue clip and rubber band were used to assist in traction, fully exposing the muscular layer. **c** After incising the muscular layer, the lesion was resected in full thickness. Extra-luminal adipose tissue was visible in the surgical area (arrow). **d** Postoperative wound surface after full-thickness resection of the lesion. **e** The wound surface of the intrinsic muscular layer was closed with tissue forceps. **f** The wound surface of the mucosal layer was closed with tissue forceps, achieving complete wound closure. **g** Observation of the serosal surface of the gross specimen, approximately 2.0 cm × 1.6 cm in size. **h** Observation of the mucosal surface of the gross specimen, approximately 2.5 cm × 2.2 cm in size.

Intermittent double-layer closure for full-thickness defect after super minimally invasive stepwise resection of early rectal cancer.Video 1

The super minimally invasive stepwise resection combined with intermittent double-layer closure technique can improve the safety of local resection for digestive tract tumors and is a feasible and effective method. By double-layer suturing of the intrinsic muscular layer and mucosal layer, it can minimize dead space to the greatest extent, potentially reduce the risk of infection, and promote healing.

Endoscopy_UCTN_Code_TTT_1AQ_2AK

## Notes

Endoscopy E-Videos https://eref.thieme.de/e-videos
*E-Videos* is an open access online section of the journal *Endoscopy* , reporting on interesting cases and new techniques in gastroenterological endoscopy. All papers include a high-quality video and are published with a Creative Commons CC-BY license. Endoscopy E-Videos qualify for HINARI discounts and waivers and eligibility is automatically checked during the submission process. We grant 100% waivers to articles whose corresponding authors are based in Group A countries and 50% waivers to those who are based in Group B countries as classified by Research4Life (see: https://www.research4life.org/access/eligibility/ ). This section has its own submission website at https://mc.manuscriptcentral.com/e-videos .
